# ElecMeter: An index for evaluating dissatisfaction of social decision-making

**DOI:** 10.1371/journal.pone.0354798

**Published:** 2026-09-11

**Authors:** Maryam Shahdoust, Zahra Eidi, Rouzbeh Torabi, Mehdi Sadeghi

**Affiliations:** 1 School of Biological Sciences, Institute For Research In Fundamental Sciences (IPM), Tehran, Tehran, Iran; 2 Pegah Dadekavan Sharif Company (TAPSELL), Tehran, Tehran, Iran; 3 Medical Genetics, National Institute of Genetic Engineering and Biotechnology, Tehran, Tehran, Iran; The University of Warwick, UNITED KINGDOM OF GREAT BRITAIN AND NORTHERN IRELAND

## Abstract

Understanding dissatisfaction in social decision-making is important for diagnosing structural features of collective outcomes that may be informative about differences in acceptance and cohesion. Existing electoral metrics typically focus on satisfaction or utility, overlooking dissatisfaction as an independent and socially meaningful factor. We introduce ElecMeter, a novel index for single-winner elections that defines voter dissatisfaction as the normalized rank of the elected candidate and incorporates both average and distributional features. A divergence-based component compares the observed distribution to benchmarks of maximal dissatisfaction, polarization, and neutrality using Jensen–Shannon divergence. Scores are aggregated via a generalized mean controlling sensitivity to extreme dissatisfaction. Simulations show that ElecMeter distinguishes electorates with similar average dissatisfaction but different distributional structures. Applied to the classic Breakfast Items dataset, ElecMeter consistently identifies winners across parameter settings and aligns with several classical voting rules, demonstrating internal robustness of the index. All code and data are available on GitHub, ensuring reproducibility.

## Introduction

Voting is one of the basic instrument in social decision-making [1,2]. In any collective decision, some voters see their preferences well represented, while others feel excluded or opposed to the outcome. Most formal assessments of voting systems emphasize satisfaction—specifically, the alignment of outcomes with voter utility, representation, or social welfare goals [3–6]. However, dissatisfaction is equally important, capturing the intensity and structure of opposition within the electorate, which can signal polarization, rejection, or instability in collective choices. For instance, the “expected social utility” quantifies the winner’s relative utility in relation to a social optimum, capturing how closely a system comes to selects the candidate that maximizes aggregate voter approval [3]. These frameworks are rooted in classical utility theory, where individuals are modeled as rational agents maximizing preferences [7–9]. However, they frequently overlook dissatisfaction [10], which may arise even when formal utility seems to be maximized. Dissatisfaction has been associated in the political behavior literature with perceptions of alienation, unfairness, or reduced acceptance of outcomese [11,12]. According to Herzberg’s two-factor theory [13], dissatisfaction is a distinct construct influenced by different causes, not only the absence of satisfaction. It is a distinct and complex response that may occur even when formal utility is maximized. Even when formal fairness standards are met, dissatisfaction may coexist with perceptions of alienation or reduced acceptance of outcomes [11,12].

Despite its importance, dissatisfaction has received limited attention in computational social choice and political science literature. Most models assume that satisfaction is the central concern, and the idea of quantifying dissatisfaction as a first-class outcome remains underdeveloped. Yet, research in political behavior suggests that dissatisfaction plays a key role in shaping civic engagement [14]. Ezrow and Xezonakis demonstrate that diminishing satisfaction with democracy associated not with apathy but with increased voting turnout, especially among opposition parties and dissatisfied subgroups [15]. This finding suggests that dissatisfaction can mobilize political change and should not be treated as a purely negative or pathological condition.

Outside political science, dissatisfaction has been studied in service quality, public sector evaluation, and behavioral economics. Evaluation theory posits that dissatisfaction occurs when perceived outcomes do not meet expectations, particularly when these expectations include emotional or moral significance. [16–18]. Unlike satisfaction, typically linked to fulfilled desires, dissatisfaction often reflects perceived violations of fairness or efficacy, a phenomenon highly relevant in elections [19].

In our view, voter dissatisfaction can be inferred directly from ranked preference data. A voter who ranks the winner first is likely satisfied, whereas one who ranks the winner last is likely dissatisfied. This ordinal relationship provides a natural and interpretable signal embedded in the preference structure. Surprisingly, most treatments of ranked data use rankings solely to compute aggregate outcomes (e.g., Borda scores or Condorcet winners [20–23])rather than to analyze the broader distributional structure of voter responses. We focus on the Borda count as a well-known aggregation method but show that, under the lens of dissatisfaction, it may fail to capture the full sentiment of the electorate.

**Fig 1:**
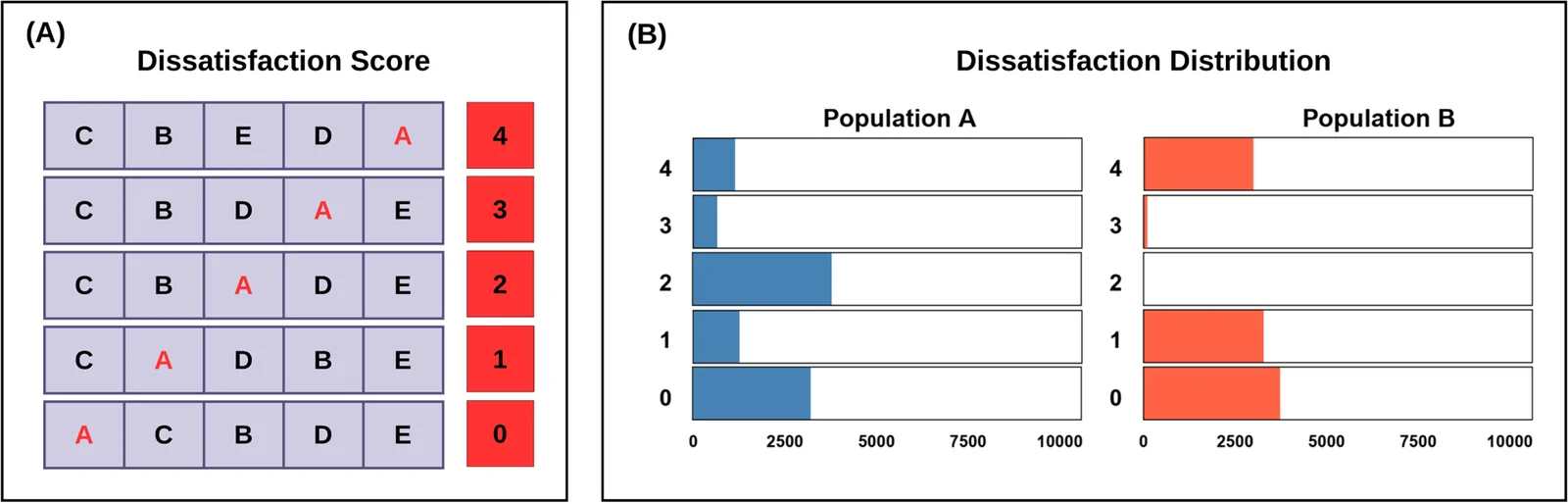
Illustration of voter dissatisfaction scoring and distribution. (A) shows how dissatisfaction scores are assigned from ranked ballots, assuming candidate A is the winner. The score equals the number of candidates ranked above the winner—ranging from 0 (most satisfied) to 4 (most dissatisfied) in a five-candidate election. (B) displays bar plots of dissatisfaction distributions for two populations: Population A: (3197, 1247, 3773, 646, 1137), Population B: (3728, 3268, 0, 8, 2996). Both populations have the same average dissatisfaction of approximately 0.382. However, their ElecMeter_3_ values differ—0.708 for Population A and 0.577 for Population B—reflecting structural differences in dissatisfaction beyond the average.

In this paper, we propose an index for measuring voter dissatisfaction in single-winner elections. The core idea is to compute a dissatisfaction score for each voter based on the rank position of the winning candidate (Fig 1A). These individual scores are then aggregated into a distribution (Fig 1B), which is compared to several benchmark distributions using the Jensen–Shannon divergence [24]. These benchmarks represent ideal, maximally dissatisfied, neutral, and polarized pattern to represent theoretically meaningful extremes and transitional states within the space of possible dissatisfaction distributions [25–28]. Each benchmark is a qualitatively distinct and clearly defined manifestation of voter dissatisfaction. They serve as theoretical benchmarks for comparing observed distributions and they are deliberately chosen theoretical reference structures selected for their interpretability, structural clarity, and analytical distinctiveness. The ideal condition refers to a scenario in which all voters are fully satisfied with the outcome, unanimously supporting the winning candidate. The maximally dissatisfied benchmark reflects a scenario in which all voters assign the winner the lowest possible rank; the neutral benchmark assumes equal distribution across all dissatisfaction levels, and the polarized benchmark concentrates mass at the extremes (fully satisfied and fully dissatisfied), reflecting a deeply divided electorate. From these divergences, we derive three intermediate ratios that capture the relative divergence from the ideal pattern compared to each reference type. The final Dissatisfaction Index, called ElecMeter, integrates the average voter dissatisfaction score with the most extreme divergence-based index via a generalized mean formulation.

While dissatisfaction is often discussed in relation to legitimacy, stability, and democratic acceptance, the present work does not seek to measure these concepts directly. Instead, we focus on dissatisfaction as a procedural and structural property of electoral outcomes, inferred from ordinal preference rankings. The proposed index is intended as a diagnostic tool for comparing collective outcomes under clearly specified assumptions, rather than as an empirical measure of voter attitudes or democratic performance.

ElecMeter is bounded by [0,1]. Since “0” means entire satisfaction and “1” complete dissatisfaction, this boundedness improves interpretability. The [0,1] range allows for comparisons across voting rules, populations, and situations.

In addition to serving as a diagnostic index, ElecMeter can be regarded as an independent voting rule. Conventional electoral systems determine winners by maximizing satisfaction (e.g., plurality, Borda, approval), while ElecMeter adopts a contrasting approach: it identifies the alternative associated with the lowest measured structural dissatisfaction under the proposed definition. In this context, ElecMeter can be interpreted as a conservative, minimax-inspired diagnostic principle for evaluating collective outcomes, in the sense that it prioritizes limiting the most extreme forms of structural dissatisfaction rather than optimizing average satisfaction. To exemplify this potential, we apply ElecMeter to the Green and Rao [29] breakfast preference data, demonstrating its capability to evaluate outcomes and inform the selection of a collective decision.

The proposed index is designed under the assumption that all voters submit complete, tie-free rankings of the candidates; that is, each voter expresses a strict preference order without abstentions or indifferences. It is further assumed that all voters are in equivalent conditions: differences in geographical location or social status are not considered, and every ballot carries the same weight. Finally, we suppose that no single voter acts as a dictator, so that electoral outcomes do not depend solely on one individual’s preference. These assumptions provide a consistent basis for mapping rank positions to dissatisfaction levels and for the theoretical analysis of the index.

An additional perspective on ElecMeter comes from statistical physics. The level of dissatisfaction illustrated in Fig 1B resembles the quantized energy levels used to describe microstates in the statistical physics of a classical many-body system composed of identical particles. This analogy also serves to support the index from an alternative standpoint. In that context, a system’s state is characterized not only by its average energy but also by how that energy is distributed across particles captured through measures like entropy [30]. Analogously, our proposed index combines the normalized average dissatisfaction (reflecting the overall “internal energy” in the electorate) with a divergence-based component that measures how the dissatisfaction distribution deviates from reference patterns, capturing structural disorder or heterogeneity. This formulation aligns with principles in sociophysics, where both consensus level and distributional polarization influence system stability [31,32].

By proposing a dissatisfaction-based framework, this work complements existing utility-based models and opens a new direction for evaluating collective outcomes. Although motivated by voting theory, the framework is general and can be applied to a wide range of social decision-making processes where preferences are expressed and aggregated, from committee choices to organizational planning and beyond. It therefore offers both theoretical value for the study of social choice and practical insights for designing decision procedures that are robust to dissatisfaction.

## Materials and methods

This study introduces a novel index, called ElecMeter, to quantify voter dissatisfaction in single-winner elections based on full preference rankings.

Our framework assumes a setting in which all voters provide complete, tie-free rankings of the candidates. That is, every voter ranks all candidates from most to least preferred without abstaining or expressing indifference (e.g., ballots like “A—-” or “AB–C” are excluded). These assumptions allow for a consistent mapping between rank positions and levels of dissatisfaction. Although such conditions may not always hold in real-world elections, they provide a clear baseline for measuring dissatisfaction and facilitate the theoretical development of the index. In addition, to ensure that dissatisfaction reflects only the distribution of preferences rather than external attributes, all voters are assumed to be in equivalent conditions. Therefore, differences in geographical location or social status are disregarded, and each ballot carries the same weight. The last conditions related to the dictatorship. No single voter determines the outcome; that is, the electoral result does not depend solely on the preference of one individual. This assumption aligns with standard axioms in social choice theory and ensures that ElecMeter evaluates dissatisfaction at the collective level rather than reflecting a single individual’s view [33].

ElecMeter combines two key components: the average dissatisfaction score across voters, and the divergence of the dissatisfaction distribution from reference (idealized) distributions. These are integrated into a unified scalar score to reflect both individual and collective dissatisfaction.

### 0.1 Voter dissatisfaction score

Let $C=\{c_{1},c_{2},...,c_{K}\}$ be the set of *K* candidates. Each voter provides a strict ranking of all candidates. Let w∈C denote the winner.

For each voter, define the dissatisfaction score $d_{i}\in \{0,1,...,K-1\}$ as the position of the winning candidate in their ranking, where 0 indicates full satisfaction (winner ranked first) and K−1 indicates complete dissatisfaction (winner ranked last). Fig 1A presents an example of how dissatisfaction scores are assigned to ballots.

The empirical distribution of dissatisfaction scores is $D=(f_{0},f_{1},...,f_{K-1})$, where $f_{j}$ denotes the number of voters ranking the winner at position *j*, and $\sum_{j=0}^{K-1}f_{j}=N$, where *N* is the population size. Fig 1B illustrates the distributions of two different populations.

Dissatisfaction is defined procedurally as the ordinal rank position of the winning candidate in each voter’s preference ranking, without reference to preference intensity or cardinal utility.

### 0.2 Reference distributions

To evaluate how dispersed or polarized the dissatisfaction is, we compare *D* against four reference distributions:

- Ideal Distribution $D_{\text{ideal}}=(N,0,0,...,0)$: all voters rank the winner first.
- Maximum Dissatisfaction $D_{\text{max}}=(0,0,...,0,N)$: all voters rank the winner last.
- Neutral Distribution $D_{\text{neutral}}=\left(\frac{N}{K},...,\frac{N}{K}\right)$: equal distribution across all positions.
- Polarized Distribution $D_{\text{polarized}}=\left(\frac{N}{2},0,...,0,\frac{N}{2}\right)$: half of voters rank the winner first, half rank them last.

### 0.3 Jensen–Shannon Divergence (JSD)

To assess how far the observed distribution deviates from these references, we compute the Jensen–Shannon divergence [24]:


$$\begin{array}{c} JS(P,Q)=\frac{1}{2}D_{KL}(P\;\|\;M)+\frac{1}{2}D_{KL}(Q\;\|\;M), \end{array}$$ (1)

where $M=\frac{1}{2}(P+Q)$, and $D_{KL}$ is the Kullback–Leibler divergence [34]:


$$\begin{array}{c} D_{KL}(P\;\|\;Q)=\sum_{j}P_{j}\log_{2}\left(\frac{P_{j}}{Q_{j}}\right), \end{array}$$ (2)

with the convention that 0log(0/0)=0. The Jensen–Shannon divergence is computed using base-2 logarithms, so that divergence values are measured in bits; this choice does not affect relative comparisons and is adopted for interpretability and consistency with standard practice.

Let $P=\frac{D_{observed}}{N}$ represent the normalized observed distribution. We define the following divergences:


$$\begin{array}{cc} J_{1} & =JS(P,\frac{D_{\text{ideal}}}{N}) \end{array}$$ (3)

$$\begin{array}{cc} J_{2} & =JS(P,\frac{D_{\text{max}}}{N}) \end{array}$$ (4)

$$\begin{array}{cc} J_{3} & =JS(P,\frac{D_{\text{neutral}}}{N}) \end{array}$$ (5)

$$\begin{array}{cc} J_{4} & =JS(P,\frac{D_{\text{polarized}}}{N}) \end{array}$$ (6)

### 0.4 Relative divergence ratios

To summarize how close the observed distribution is to each reference, we define three normalized sub-indexes:


$$\begin{array}{ccc} I_{1} & = & \frac{J_{1}}{J_{1}+J_{2}}\;\text{(Ideal vs. Maximum)} \end{array}$$ (7)

$$\begin{array}{ccc} I_{2} & = & \frac{J_{1}}{J_{1}+J_{3}}\;\text{(Ideal vs. Neutral)} \end{array}$$ (8)

$$\begin{array}{ccc} I_{3} & = & \frac{J_{1}}{J_{1}+J_{4}}\;\text{(Ideal vs. Polarized)} \end{array}$$ (9)

Then, We define the divergence-based component of the final index as:$I_{\max}=\max(I_{1},I_{2},I_{3})$.

### 0.5 Final dissatisfaction index

Let $S=\frac{1}{N}\sum_{j=1}^{K-1}f_{j}\times j$ be the average dissatisfaction score, normalized to the range [0, 1], by $S_{norm}=\frac{S}{\left(K-1\right)}$. For simplicity, we refer to this normalized value simply as *S* throughout the paper. We define the final index as a generalized mean of *S* and $I_{\max}$:


$$\begin{array}{c} {\text{ElecMeter}}_{p}={\left(S^{p}+I_{\max}^{p}\right)}^{1/p}, \end{array}$$ (10)

for a chosen $p\in {\mathbb{R}}^{+}$. Since the value of ${\text{ElecMeter}}_{p}$ tends to increase with *p*, we normalize the index by dividing it by 2^1/*p*^. This normalization bounds the index within the interval [0, 1], where values close to 0 indicate broad voter satisfaction (the winner is generally ranked highly across the electorate), and values close to 1 indicate widespread dissatisfaction (the winner is consistently ranked low). Thus, lower ElecMeter values correspond to more favorable outcomes, while higher values reflect elections that produce greater collective dissatisfaction. Therefore, it is possible to compare values not only across different choices of *p*, but also across different populations and electoral conditions in a consistent and interpretable manner:


$$\begin{array}{c} {\text{ElecMeter}}_{p}^{\text{norm}}=\frac{(S^{p}+I_{\max}^{p}{)}^{1/p}}{2^{1/p}}. \end{array}$$ (11)

In this paper, we examine several values of *p*, including *p* = 2 (Euclidean norm), and *p* = 4 (emphasizing extremes). We focus primarily on *p* = 3, as it strikes a balance between emphasizing highly dissatisfied populations (via $I_{\max}$) and maintaining sensitivity to the general satisfaction level *S*. This setting consistently provided clearer separation between groups in clustering-based evaluations and demonstrated good discriminatory power across simulated and real preference distributions.

The index is flexible and can be adapted to different normative priorities, such as equity, polarization, or consensus, depending on the electoral context or policy decision under consideration.

In addition, the ElecMeter index can be employed as a criterion to identify the most representative candidate. Given a set of rankings provided by all participants, each candidate was considered in turn as the potential winner. For each case, the ElecMeter index was calculated to summarize the overall level of dissatisfaction across participants. The candidate associated with the lowest ElecMeter index was then selected as the optimal winner. This procedure enables a systematic prioritization of candidates based on collective preferences. The Results section presents an application of the ElecMeter index to identify the winner in an election using real data [29].

### 0.6 Simulation setup

To assess the behavior and robustness of the proposed Dissatisfaction Index, we simulated 1,000 synthetic elections, each with *K* = 5 candidates and a voter population of *N* = 10,000. In each simulation, we generated frequencies of voters’ complete rankings across all possible permutations of the five candidates. The frequencies were drawn from a multinomial distribution [35], with probability profiles sampled from a Dirichlet distribution [36,37] whose concentration parameters were randomly chosen. This procedure ensures variability in voter preferences while preserving the total number of votes in each election, thereby capturing a broad spectrum of plausible electoral scenarios, including high consensus, strong polarization, and moderate diversity in preferences. The election winner in each population was determined using the Borda count method [38]. We then computed the Dissatisfaction Index and its components for each simulated election.

In addition, we conducted a sensitivity analysis to examine the behavior of ElecMeter under varying conditions. First, we varied the number of candidates while fixing the electorate size at *N* = 10,000, simulating elections with *K* = 3, 4,5 and 7, each repeated for 1,000 populations. Next, we varied the electorate size while fixing the number of candidates at *K* = 5, repeating the simulation steps for *N* = 10,000, 1,000, 100, and 10, again with 1,000 populations in each case.

The simulation code in R, as well as the different results, are publicly available at GitHub.

## Results

After simulating the vote frequencies for each candidate across all populations, we applied the Borda count voting system to determine the winner in each population (Borda count method is described in the Supplementary File). Based on the winning candidate, we then computed the dissatisfaction distribution for each population and evaluated it using the ElecMeter index (equation 11). The complete set of simulation results is provided in the file Total_Results(k = 5), available in the GitHub repository. In addition, the outcomes of alternative voting systems, including Schulze [39], Copeland [40], and plurality [41], are analyzed and documented in the file Voting.Methods_total within the same repository. Nevertheless, the focus of this paper is primarily on the Borda count method.

Table 1 summarizes some representative populations with varying dissatisfaction profiles, we selected to illustrate the behavior and interpretability of ElecMeter. It is important to note that the ElecMeter values and normalized average dissatisfaction (*S*) never reach 1, even in the most extreme cases. This is because we compute dissatisfaction only for the winner, that is, even when voters rank the winner poorly, the winning candidate signifies a baseline of collective endorsement, preventing complete maximal dissatisfaction. In the simulations, the maximum observed value of dissatisfaction is around 0.82 for population number 777 in Table 1. These values reflect the trade-off between the average dissatisfaction and divergence from ideal reference distributions. The whole results including the value of all the JSD divergence from the benchmarks are provided in the file Total_Results(k = 5) at GitHub.

**Table 1:** Selected simulated populations.

Pop	Winner	F_0_	F_1_	F_2_	F_3_	F_4_	S	$I_{max}$	ElecMeter(*p* = 2)	ElecMeter(*p* = 3)	ElecMeter(*p* = 4)
121	D	4939	30	9	3021	2001	0.427	0.611	0.527	0.535	0.542
147	D	92	9600	308	0	0	0.255	0.648	0.492	0.524	0.548
185	B	6	7937	0	2041	16	0.353	0.699	0.554	0.578	0.597
226	B	12	5705	4025	247	11	0.364	0.745	0.586	0.614	0.636
361	A	2105	1945	2059	2042	1849	0.490	0.999	0.787	0.823	0.852
416	D	5642	1252	3097	9	0	0.187	0.487	0.369	0.394	0.412
446	D	3197	1247	3773	646	1137	0.382	0.869	0.671	0.708	0.737
527	E	3728	3268	0	8	2996	0.382	0.690	0.557	0.577	0.593
608	D	8	6587	2908	475	22	0.348	0.754	0.587	0.617	0.641
693	C	8038	101	0	0	1861	0.189	0.556	0.415	0.447	0.469
777	A	2060	2148	1917	1893	1982	0.490	0.999	0.787	0.823	0.852
784	A	2707	1374	3574	785	1560	0.427	0.919	0.717	0.753	0.782

*Pop* denotes the population number; *Winner* indicates the selected candidate (A, B, C, D, or E); F_0_ to F_4_ represent the frequency distribution across the five levels of dissatisfaction; *S* is the normalized average dissatisfaction; $I_{max}$ is the maximum normalized divergence from benchmark distributions; ElecMeter(*p* = 2), ElecMeter(*p* = 3), and ElecMeter(*p* = 4) denote the ElecMeter values computed using *p* = 2, 3, 4, respectively.

**Fig 2:**
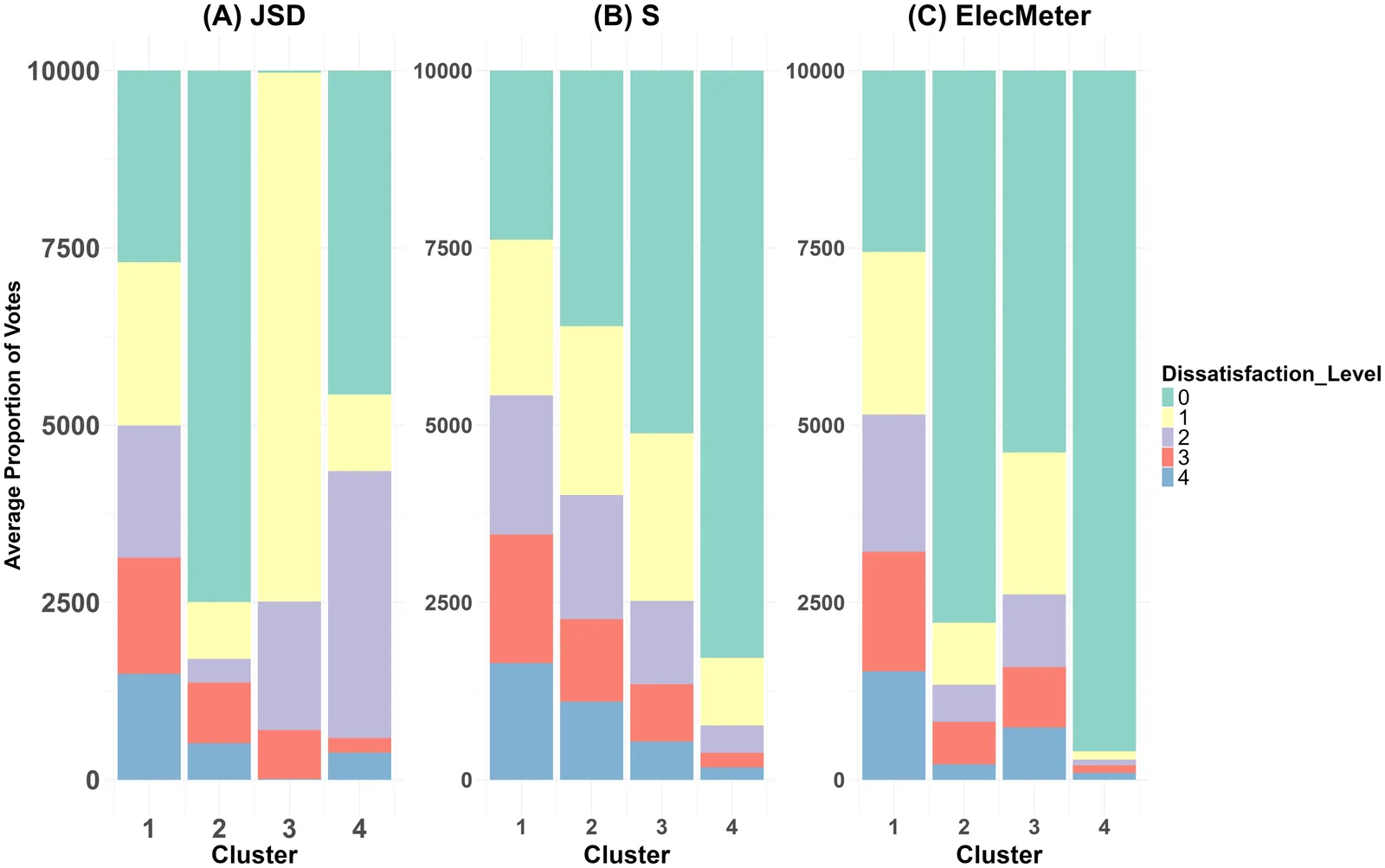
Average dissatisfaction profiles within clusters obtained under three alternative clustering criteria. Panels (A)–(C) display, for each cluster, the mean proportion of voters at each dissatisfaction level. In panel (A), populations are clustered based on the Jensen–Shannon divergence (JSD) between their dissatisfaction distributions, capturing similarities in distributional shape. In panel (B), clustering is based on the Euclidean distance between normalized average dissatisfaction values (*S*), reflecting differences in overall dissatisfaction magnitude. In panel (C), populations are clustered using the Euclidean distance between their ElecMeter values, which integrate both average dissatisfaction and divergence from reference distributions.

To evaluate whether ElecMeter meaningfully captures patterns of collective dissatisfaction, we first applied hierarchical clustering [42] to the 1,000 simulated populations using two alternative distance criteria: (i) JSD between dissatisfaction distributions, and (ii) the Euclidean distance between *S*. Hierarchical clustering was chosen for its ability to reveal similarity structures without imposing parametric assumptions or a predefined number of clusters, making it well suited for exploratory comparison of dissatisfaction profiles. For further details on the clustering methodology and the dendrograms used to select the number of clusters, please refer to the Figure S1 in S1 Supporting Information. We next applied hierarchical clustering based on the Euclidean distance between ElecMeter values. Table 2 and Fig 2 display the average dissatisfaction distributions across the clusters formed using three clustering criteria.

**Table 2:** Mean number of voters at each dissatisfaction level across clusters.

Method	Cluster	0	1	2	3	4	Chi2-test
JSD	1	2703	2303	1863	1639	1492	<0.001
	2	7494	802	333	855	515	
	3	29.5	7457	1810	691	12.2	
	4	4567	1081	3764	204	385	
S	1	2384	2194	1961	1816	1645	<0.001
	2	3604	2380	1750	1164	1102	
	3	5115	2364	1175	805	540	
	4	8281	952	385	207	174	
ElecMeter	1	2557	2293	1936	1685	1530	<0.001
	2	7786	876	521	598	219	
	3	5385	2002	1025	851	737	
	4	9599	116	81.6	108	95.6	

Method indicates three different methods for clustering. Columns labeled 0–4 correspond to dissatisfaction levels (0 = fully satisfied, 4 = fully dissatisfied). Chi2-test displays the result of chi-squared tests assessing whether distributions differ significantly across clusters. Results confirm statistically significant differences at the 5% level.

Applying hierarchical clustering directly to the ElecMeter values (*p* = 3) yielded four clusters with distinct dissatisfaction profiles (Table 2 and Fig 2C). Comparisons with clusters formed using JSD and *S* reveal that clustering based on ElecMeter (Fig 2C) generates groups that are coherent in terms of both the mean level of dissatisfaction and the underlying distributional structure. Populations within the same ElecMeter-based cluster share similar average dissatisfaction while also exhibiting comparable distributional profiles, a distinction not fully captured when clustering is based solely on *S* or JSD.

According to Table 3 revealing the mean and standard deviation of the index in the clusters, cluster 1 contained populations with the highest normalized average dissatisfaction (*S*) and maximum divergence ($I_{\max}$), reflecting strongly dissatisfied structures. Cluster 4, in contrast, represented broadly satisfied populations with both *S* and $I_{\max}$ close to zero. Clusters 2 and 3 exhibited intermediate dissatisfaction patterns, with Cluster 2 closer to the satisfied end of the spectrum and Cluster 3 showing more balanced mid-level dissatisfaction.

**Table 3:** Summary statistics of dissatisfaction measures across simulated population clusters.

Method	Cluster	S		$I_{\max}$		ElecMeter(*p* = 3)	
		Mean	SD	Mean	SD	Mean	SD
JSD	1	0.423	0.072	0.933	0.110	0.763	0.092
	2	0.152	0.110	0.350	0.216	0.287	0.177
	3	0.330	0.050	0.712	0.049	0.583	0.043
	4	0.269	0.082	0.566	0.131	0.466	0.109
S	1	0.453	0.026	0.976	0.039	0.799	0.032
	2	0.344	0.028	0.817	0.093	0.665	0.071
	3	0.232	0.041	0.594	0.131	0.481	0.102
	4	0.076	0.039	0.219	0.118	0.176	0.094
ElecMeter	1	0.433	0.054	0.951	0.073	0.778	0.061
	2	0.115	0.052	0.281	0.074	0.229	0.061
	3	0.239	0.058	0.575	0.080	0.468	0.065
	4	0.024	0.019	0.065	0.042	0.053	0.035

The Methods column indicates the feature used to compute similarities between populations: JSD is the Jensen–Shannon divergence between population distributions; *S* is the Euclidean distance between the normalized average of distributions; and ElecMeter is the Euclidean distance between the ElecMeter values of population distributions. Columns report the mean and standard deviation (SD) for each measure: *S* = normalized average dissatisfaction; $I_{\max}$ = maximum normalized divergence from benchmark distributions; ElecMeter(*p* = 3) = ElecMeter values computed with *p* = 3.

Table 4 presents the contingency table comparing the resulting cluster memberships. If *S* and JSD were strongly aligned, we would expect most populations to fall along the diagonal of the table, indicating consistent assignments across the two methods. However, the table shows substantial off-diagonal counts, highlighting that the two measures partition the populations differently. This divergence reflects the distinct aspects each measure captures: *S* quantifies the magnitude of dissatisfaction without regard to its distributional shape, whereas JSD emphasizes the structural differences between dissatisfaction profiles regardless of their overall level. Consequently, *S* may group together populations with similar mean dissatisfaction but contrasting distributional patterns, while JSD separates them.

**Table 4:** Contingency tables comparing cluster assignments obtained from different methods.

	*S*			
JSD	1	2	3	4
1	710	107	70	2
2	4	6	28	50
3	0	3	1	0
4	1	6	10	2
	ElecMeter			
JSD	1	2	3	4
1	839	1	49	0
2	6	45	23	14
3	3	0	1	0
4	5	3	11	0
	ElecMeter			
*S*	1	2	3	4
1	713	0	2	0
2	111	0	11	0
3	29	11	69	0
4	0	38	2	14

The first table compares clusters obtained using *S* and JSD, showing that the two measures do not always produce the same grouping, reflecting that each captures a different aspect of dissatisfaction. The second table compares clusters from ElecMeter and JSD, showing a stronger alignment for highly dissatisfied (JSD Cluster 1) and highly satisfied populations (JSD Cluster 2), though mismatches exist in intermediate profiles. The third table compares ElecMeter and *S*, revealing that ElecMeter further differentiates populations within the same *S* category by incorporating structural divergence information.

Comparisons with the JSD-based clusters revealed both agreements and differences (Table 4). For example, the most dissatisfied populations (Cluster 1 in both methods) aligned closely, as did the most satisfied populations, though the latter appeared as Cluster 2 in the JSD solution and Cluster 4 in the ElecMeter solution. Other clusters showed mismatches: several mixed or mid-dissatisfaction populations grouped together in the JSD-based clustering were split by ElecMeter according to their divergence patterns. A similar comparison with the *S*-based clusters (Table 4) confirmed that ElecMeter captures patterns not reflected by average dissatisfaction alone. While *S*-based clustering mainly separated populations along a monotonic satisfaction–dissatisfaction gradient, ElecMeter further discriminated between populations with comparable *S* values but differing internal distribution shapes. In addition, to quantify the agreement between clustering solutions, we computed the Adjusted Rand Index (ARI) [43], which measures the similarity between two partitions while correcting for chance. Consistent with the contingency tables, the ARI between JSD- and S-based clustering was relatively low (0.387), indicating substantial differences in assignments and confirming that the two criteria capture distinct aspects of dissatisfaction. The ARI between JSD- and ElecMeter-based clustering was higher (0.673), showing that ElecMeter preserves much of the structural separation identified by JSD. Similarly, the ARI between S- and ElecMeter-based clustering was moderate (0.538), reflecting that ElecMeter also retains information about average dissatisfaction. These results support the conclusion that ElecMeter integrates complementary information from both measures rather than simply replicating one of them.

**Fig 3:**
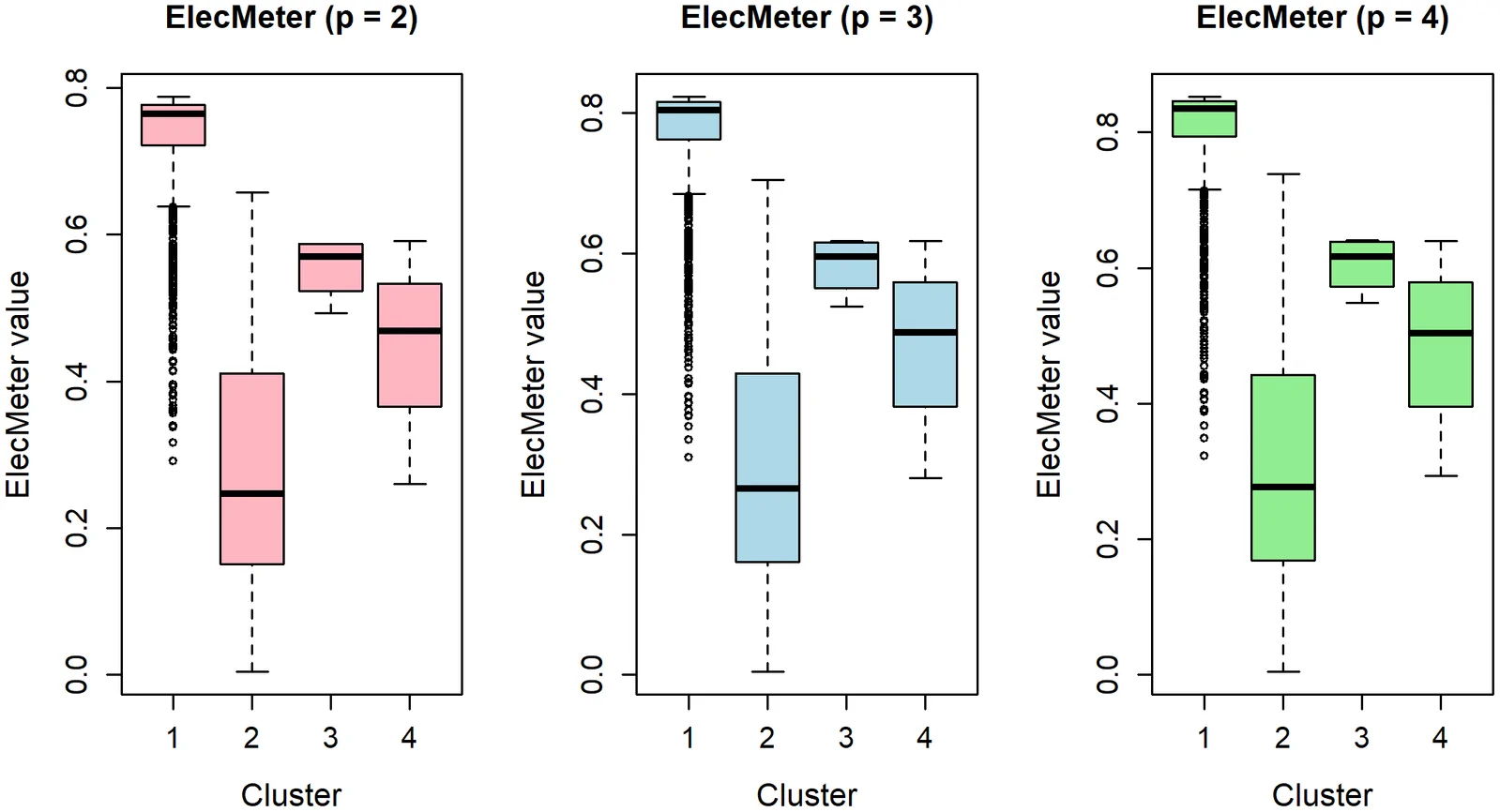
Boxplots of the ElecMeter index across the four identified clusters based JSD between populations dissatisfaction distribution, computed using different values of the power parameter p∈{2,3,4}, each normalized by 2^1/*p*^. The index reveals distinct patterns of dissatisfaction corresponding to the cluster structure, with higher values indicating more widespread or extreme voter dissatisfaction.

As the ElecMeter index includes a tunable power parameter *p* (equation 11), selecting an appropriate value is important for balancing sensitivity to dissatisfaction extremes with robustness to distributional variation. To guide this choice, we computed ElecMeter values for p∈{2,3,4} and examined their distributions across the JSD-based clusters (Fig 3). Across all three parameter settings, the relative ordering of median ElecMeter values among clusters was consistent, with Cluster 1 showing the highest values and Cluster 2 the lowest. The separation between intermediate clusters was more pronounced for *p* = 3 and *p* = 4, with *p* = 3 offering a balance between discrimination of structural patterns and interpretability. Based on this, we selected *p* = 3 as the primary parameter setting for subsequent analyses. To confirm that our choice of *p* = 3 in ElecMeter balances both the magnitude and the structure of dissatisfaction, we also examined the distribution of ElecMeter values across clusters obtained from *S*-based hierarchical clustering (see Figure S2 in the S1 Supporting Information file).

The differences between *p* values were minimal in this setting, as *S* is an intrinsic component of the index. This indicates that ElecMeter reliably preserves the magnitude ordering of dissatisfaction when such differences dominate the clustering. However, because the separation in *S*-based clustering is expected by design, it serves primarily as a validation check, whereas parameter selection for *p* is better guided by the JSD-based clustering, which emphasizes structural differences in dissatisfaction patterns.

**Fig 4:**
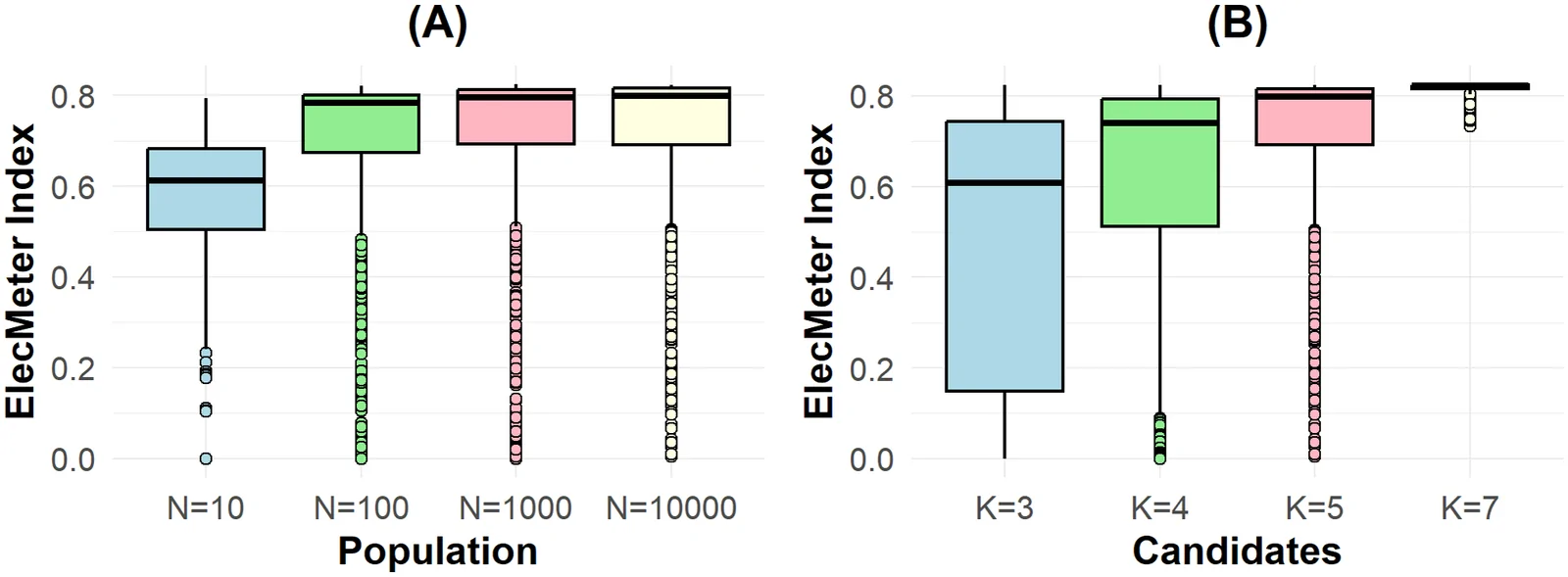
Dissatisfaction distributions across varying population sizes and candidate numbers. (A) Boxplots showing the distribution of dissatisfaction levels for varying population sizes (*N*), with the number of candidates fixed at *K* = 5. (B) Boxplots showing the distribution of dissatisfaction levels for varying numbers of candidates (*K*), with the population size fixed at *N* = 10,000. For all the analysis the parameter*P* to compute the Dissatisfaction Index is set as three.

To further evaluate the robustness of the proposed Dissatisfaction Index, we conducted sensitivity analyses by varying the size of the electorate and the number of candidates (Fig 4). Fig 4A illustrates the distribution of ElecMeter values across simulations with different electorate sizes (*N* = 10, 100, 1,000, 10,000) while fixing the number of candidates at *K* = 5. For small electorates (*N* = 10), the distribution of the index is highly dispersed, reflecting the strong influence of sampling variability in small populations. As *N* increases, the spread of values narrows and the medians stabilize, indicating that ElecMeter converges toward stable estimates with larger populations. This pattern confirms that the index is well-behaved under scaling and becomes more reliable as the number of voters grows. Fig 4B shows the distribution of ElecMeter values for elections with different numbers of candidates (*K* = 3, 4, 5) while fixing the electorate size at *N* = 10,000. Here, we observe that the median value of the index increases with the number of candidates, and the interquartile range narrows. With only three candidates, elections can produce both very low and very high dissatisfaction, leading to a wider spread. By contrast, as the number of candidates grows, the distribution of dissatisfaction becomes more concentrated at higher index values, suggesting that the presence of more alternatives tends to increase the structural dissatisfaction of the electorate. Together, these results show that ElecMeter is sensitive to both the electorate size and the number of candidates in ways that align with intuitive expectations about how these factors influence dissatisfaction. The index remains robust in large populations and meaningfully captures the increase in dissatisfaction that arises when more candidates compete.

### 0.7 Illustration: Breakfast items

The Breakfast Items dataset is used here as a pedagogical and illustrative example, chosen for its transparency and frequent use in the social choice literature, rather than as a comprehensive empirical validation of the proposed index. The dataset originates from the study by Green and Rao [29], in which 42 respondents (21 Wharton MBA students and their wives) provided strict preference rankings over 15 sweet breakfast items under six different contexts. The rankings for each context are available in separate files within the PrefLib collection. In this illustration, we focus on the “Overall preferences” dataset.

We applied ElecMeter by computing dissatisfaction levels and corresponding index values for each candidate item, and then identified the item with the lowest ElecMeter value as the most representative winner. The analysis was conducted for multiple parameter values (*p* = 2, 3, 4, 5). Across all values of *p*, the same candidate was consistently identified as the winner. Pairwise correlations between ElecMeter values were high (r=0.79−−0.96), indicating that the relative ranking of candidates is robust to the choice of *p*. Notably, the minimum ElecMeter value remained above 0.5, suggesting that even the most representative item did not fully satisfy all participants.

For contextual comparison, we also applied several classical voting rules, including Borda count [20,21,38,44,45], Dowdall [46,47], Schulze [39], Instant-Runoff [48], and Plurality [22], to the same dataset. All methods selected the same winner as ElecMeter. This agreement highlights the consistency of the ElecMeter approach with traditional voting rules, while ElecMeter additionally provides an explicit quantitative measure of dissatisfaction.

All detailed results, including ElecMeter values for all candidates, correlation matrices, and winners under each voting rule, are reported in Table S3 and Table S4 in S1 Supporting Information and are publicly available at GitHub.

## Discussion

The primary aim of this study was to develop a robust index to quantify voter dissatisfaction resulting from a social choice, such as a single-winner election. Existing measures in computational social choice and political science largely focus on satisfaction or utility-based outcomes, often overlooking dissatisfaction as an independent and socially significant phenomenon [3,10,14]. Our approach addresses this gap by introducing the ElecMeter index, which is designed to capture both the magnitude and the structure of dissatisfaction in the electorate. Beyond elections, the proposed index is intended as a general tool for quantifying dissatisfaction in social choice, with potential applications ranging from small-scale settings, for example, a workplace decision on uniform colors, to large-scale political elections, as well as survey-based contexts where respondents provide ordinal rankings or ratings of a set of options, provided the defined conditions are met. The assumptions adopted in this study, such as complete rankings and equal weight for all voters, primarily serve to simplify the theoretical development and to make the index transparent in its initial presentation. These conditions are not intrinsic limitations of the approach; rather, they provide a baseline from which future extensions can relax assumptions to better approximate real-world electoral and decision-making environments.

The ElecMeter index(equation 11) integrates two complementary components of voter dissatisfaction: the normalized average dissatisfaction score (*S*) and the divergence-based component ($I_{\max}$). This twofold sensitivity addresses a major limitation of metrics based solely on averages, which can obscure important structural differences in dissatisfaction patterns. To integrate both average and structural dissatisfaction into a single scalar measure, we use a generalized mean of degree *p* (equation 11). The power parameter *p* allows the index to be tuned for different applications: larger values of *p* place greater emphasis on extreme dissatisfaction, whereas smaller values balance the two components more evenly. This approach is analogous to norm-based aggregation used in multi-objective optimization [49] and robustness analysis. Our simulation study demonstrates that by integrating the normalized average dissatisfaction score with a divergence-based component, ElecMeter captures differences in concentration, dispersion, and polarization that are not reflected by the mean alone. Simulations were conducted using the Borda rule as a representative ranking-based voting method, but the proposed framework is not restricted to this voting rule.

An important advantage of the ElecMeter formulation is that it is bounded within the unit interval [0,1]. This boundedness enhances interpretability, since the values have a clear absolute meaning: 0 corresponds to complete satisfaction and 1 to complete dissatisfaction. The [0,1] range facilitates direct comparisons across different voting rules, populations, and various conditions. Moreover, the bounded scale allows ElecMeter to serve as a standardized metric, making it suitable not only for within-study analysis but also for meta-analyses or empirical applications where consistent scaling is essential.

A key design choice in ElecMeter is the use of Jensen–Shannon divergence(JSD) [24] to quantify how far the observed dissatisfaction distribution lies from each benchmark, ensuring both flexibility and interpretability. In our framework, the maximally dissatisfied, polarized, and neutral distributions are treated as three representative benchmark structures, each capturing a qualitatively distinct and well-defined form of voter dissatisfaction. The maximally dissatisfied benchmark places all voters at the highest dissatisfaction level; the polarized benchmark concentrates voters at both extremes of satisfaction and dissatisfaction; and the neutral benchmark distributes dissatisfaction evenly across all levels (a uniform distribution of dissatisfaction levels). The term “neutral” is used here in a purely descriptive sense to denote a uniform distribution of dissatisfaction levels, and should not be interpreted as normatively neutral or politically desirable. These benchmarks are not intended to exhaustively enumerate all possible dissatisfaction configurations, but rather to serve as deliberately chosen theoretical reference points against which observed distributions can be compared [25–28]. Alternative patterns, such as right-skewed or bimodal distributions, can be understood as special cases or less extreme manifestations of these benchmarks. For instance, the polarized pattern in our model corresponds to a bimodal structure, with voters clustered at both the fully satisfied and fully dissatisfied extremes. Similarly, a strongly right-skewed distribution can, in its extreme form, be identical to the maximally dissatisfied pattern, with the majority of voters at the highest dissatisfaction level. We do not introduce these alternatives as separate benchmarks because they are less precisely defined and would require additional parameters (e.g., degree of skewness or modal separation), whereas the three benchmarks used here are fully specified and interpretable without such assumptions.

ElecMeter is conceptually related to existing measures of polarization, inequality, and dispersion, including entropy-based indices and classical polarization measures such as those of Esteban–Ray and Wolfson [50,51]. These approaches typically quantify heterogeneity or polarization in cardinal distributions (e.g., income, ideology, or group size) and do not rely on explicit electoral reference outcomes. Relatedly, entropy-based measures have been proposed to assess dissatisfaction in electoral settings by focusing on the dispersion of collective outcomes rather than on individual-level satisfaction metrics [52]. By contrast, ElecMeter is designed as a benchmark-based index for ordinal electoral data, evaluating how observed dissatisfaction distributions,derived from voters’ rank orderings of the winning alternative,relate to a small set of interpretable reference configurations (ideal, maximally dissatisfied, neutral, and polarized). Accordingly, ElecMeter should be viewed as complementary to these measures rather than a substitute, addressing a distinct analytical objective focused on the structure of dissatisfaction induced by a specific electoral outcome.

For each benchmark, we normalize the JSD from the observed distribution by its divergence from the ideal distribution, yielding interpretable sub-indices (*I*_1_, *I*_2_, *I*_3_ in equations 7, 8, 9) that are directly comparable across benchmarks. To aggregate them, we considered several options—the arithmetic mean, the geometric mean, and the absolute range (|max−min|)—and found that all produce index values within [0,1] that are highly correlated with one another (See “Sensitivity to Aggregation Rule” in S1 Supporting Information). Thus, the overall conclusions of ElecMeter are robust to the aggregation rule. We adopt the maximum value ($I_{\max}$) because it follows a conservative minimax principle: if dissatisfaction strongly resembles any benchmark pattern (maximal divergence, neutrality, or polarization), this structure should dominate the assessment. Averaging rules can mask such sharp divergences, while $I_{\max}$ preserves interpretability, each sub-index retains its clear meaning, and the maximum highlights the dominant dissatisfaction pattern for a given election. This choice also allows ElecMeter to separate populations with similar average dissatisfaction but very different structural forms, which is a core motivation for its design. We acknowledge that the use of $I_{\max}$ reflects a deliberate normative design choice aligned with the goal of identifying dominant structural dissatisfaction patterns, rather than a claim of universal optimality. Alternative aggregations may be preferable in settings that prioritize smoother sensitivity across multiple divergence dimensions.

The distinction between average dissatisfaction and structural dissatisfaction is illustrated by the two simulated populations shown in Table 1 (populations 446 and 527). Although both electorates comprise 10,000 voters and have the same normalized average dissatisfaction (*S* = 0.382), their dissatisfaction distributions differ substantially. Population 446 exhibits a distribution closer to the neutral benchmark, whereas population 527 is more concentrated at the lowest and highest dissatisfaction levels. Consequently, ElecMeter assigns different index values (0.708 and 0.577, respectively), reflecting their distinct structural divergence from the benchmark distributions. This example demonstrates that incorporating the divergence-based component enables ElecMeter to distinguish electorates that appear identical when only average dissatisfaction is considered. Conversely, when dissatisfaction distributions are structurally similar, ElecMeter produces comparable values, indicating that the index does not artificially exaggerate differences among electorates with similar dissatisfaction profiles. Additional examples in Table 1 further demonstrate that ElecMeter consistently distinguishes structurally different dissatisfaction patterns while remaining stable for similar ones. Indeed, ElecMeter is not redundant with either its normalized average dissatisfaction component (*S*) or its divergence-based component ($I_{\max}$); rather, it synthesizes both into a single measure. This is evident from the clustering analysis (Fig 2). Clustering based on JSD (Fig 2A) primarily groups populations according to the shape of their dissatisfaction distributions, whereas clustering based on *S* (Fig 2B) reflects differences in overall dissatisfaction magnitude while largely ignoring structural heterogeneity. In contrast, clustering based on ElecMeter (Fig 2C) simultaneously captures both dimensions. This interpretation is supported by the contingency analysis (Table 4) and the adjusted Rand indices. The moderate agreement between the JSD- and *S*-based partitions (ARI = 0.39) indicates that structural divergence and average dissatisfaction describe complementary aspects of electoral outcomes. As expected, ElecMeter shows stronger agreement with the JSD-based clustering (ARI = 0.67) than with the *S*-based clustering (ARI = 0.54), reflecting the contribution of the divergence-based component while still retaining sensitivity to average dissatisfaction. Importantly, ElecMeter does not simply reproduce either partition. Extreme dissatisfaction profiles remain largely consistent across clustering methods, whereas populations with intermediate dissatisfaction are further differentiated according to the joint influence of average dissatisfaction and structural divergence. These findings confirm that ElecMeter provides an integrated characterization of electoral dissatisfaction by combining both magnitude and distributional structure within a single interpretable framework.

The sensitivity analysis (Fig 4) further demonstrates the robustness of ElecMeter. As the electorate size increases from *N* = 10 to *N* = 10,000, the variability of the index decreases and its distribution converges toward stable values, indicating that ElecMeter is not unduly sensitive to sample size and remains reliable across both small- and large-scale elections (Fig 4A). At a fixed electorate size (*N* = 10,000), increasing the number of candidates (*K* = 3,4,5, and 7) systematically increases the index values (Fig 4B), reflecting the greater potential for voter dissatisfaction in more competitive electoral settings. This trend is consistent with theoretical expectations, as a larger candidate pool reduces the likelihood that the winning candidate coincides with an individual voter’s top preference [53,54]. Overall, these results indicate that ElecMeter is both robust to changes in sample size and responsive to meaningful characteristics of the electoral setting.

ElecMeter can also be used as an exploratory or complementary decision-support criterion for assessing electoral outcomes. In this setting, one may explore identifying the candidate whose election minimizes the ElecMeter score, thereby favoring outcomes associated with lower structural dissatisfaction among voters. In the illustrative application using the Breakfast Items dataset [29], we demonstrated how dissatisfaction-based scores can be computed for each candidate and used to identify a representative outcome. The candidate minimizing the ElecMeter index coincided with the winners selected by several classical voting rules, including Borda count [20,21,38,44,45], Dowdall [46,47], Schulze [39], Instant-Runoff [48], and Plurality [22]. This alignment illustrates the internal consistency of the ElecMeter framework, while highlighting its distinctive contribution: unlike standard voting rules, it explicitly characterizes the distribution of voter dissatisfaction rather than focusing solely on aggregate rankings. Importantly, the empirical application presented here is intentionally limited in scope. The Breakfast Items dataset involves a small number of voters and alternatives, does not represent a real political election, and exhibits broad agreement across standard voting rules, all of which constrain the discriminative power of any evaluation metric. Accordingly, this example should not be interpreted as evidence of empirical superiority or practical dominance of ElecMeter over existing methods. Instead, its purpose is to illustrate how dissatisfaction distributions are constructed, how they are compared to theoretical reference benchmarks, and how the resulting index behaves in a well-understood setting. In more complex or contested electoral environments—such as those involving larger electorates, asymmetric preference structures, or divergent winners across voting rules—the diagnostic value of ElecMeter may be more pronounced. A systematic empirical assessment in such contexts is left for future research.

We also explored the potential of ElecMeter as a comparative tool for evaluating alternative voting rules. We applied several common voting methods, including Condorcet [23], Copeland [40], Kemeny [55], Schulze [39], Instant-Runoff [48], and Plurality [22], to the simulated populations and computed ElecMeter values with *p* = 3 for each. Full results are available in the voting.Methods_Total file on GitHub. Condorcet and Copeland rules produced the lowest average ElecMeter values, consistent with their emphasis on majority consistency and minimization of pairwise dissatisfaction, whereas plurality-based methods yielded the highest dissatisfaction levels, reflecting their tendency to disregard preference rankings beyond the top choice. These results illustrate that ElecMeter can serve as a comparative diagnostic for evaluating the dissatisfaction generated by different decision-making rules.

Besides its social choice interpretation, ElecMeter can also be understood through an analogy with statistical mechanics. Similar analogies have been used in sociophysics to study voting and opinion dynamics, where insights from physics illuminate phenomena such as consensus formation, polarization, and phase transitions in social systems [31,32,56,57]. The conceptual tools of statistical mechanics offer a powerful analogy for understanding voter dissatisfaction in elections, complementing traditional approaches in political science and sociophysics [31,32]. In a classical many-body system of identical particles with quantized energy levels, the “internal energy” is defined as $U=\sum_{i}n_{i}\varepsilon_{i}$, that is, the weighted sum of energy levels by their occupation numbers. In our sociophysical analogy, the particles represent individuals, the energy levels correspond to discrete dissatisfaction states, and the internal energy reflects the aggregate dissatisfaction of society in an electoral context. To capture not only the amount but also the “organization” of this dissatisfaction, we complement *U* with an entropy-like quantity based on the JSD between the empirical distribution of dissatisfaction and benchmark distributions. Low divergence signals order and cohesion, while high divergence signals disorder and polarization. In our framework, the voting system corresponds to a single realized microstate,the observed distribution of voter dissatisfaction,rather than a statistical ensemble of all possible configurations. To adapt statistical physics concepts to this setting, we treat the empirical normalized frequencies of dissatisfaction levels as effective probabilities within this realized distribution. Taken together, these two ingredients define what we term the ElecMeter index, which plays a role directly analogous to free energy in statistical physics [30]. Just as physical systems are stabilized by minimizing free energy, electoral systems with a lower ElecMeter are interpreted as exhibiting lower aggregate dissatisfaction and more cohesive dissatisfaction structures, within the confines of the model.

## Conclusion

This study introduced ElecMeter, a novel diagnostic index that integrates both the magnitude and structure of voter dissatisfaction. The index is bounded between 0 and 1, enabling intuitive interpretation and direct comparisons across elections. Through simulations and clustering analyses, we showed that ElecMeter distinguishes electorates with similar average dissatisfaction but different structural profiles, such as polarization or fragmentation. While the current formulation relies on simplifying assumptions (complete rankings, equal weights, and single-winner settings), these provide a baseline for future extensions to incomplete ballots, variable turnout, and multi-winner systems. By capturing dissatisfaction as both a matter of degree and of structure, ElecMeter offers a framework for evaluating dissatisfaction structures in collective decisions across contexts ranging from small organizations to national elections, within the confines of the proposed framework. Moreover, ElecMeter can serve not only as a descriptive measure but also as a potential decision-support framework in social choice settings.

## Supporting information

S1 Supporting Information Supplementary figures, tables, additional methodological details, and supplementary results supporting the findings presented in this study. (PDF)

## References

[1] Becker R. Voting behavior as social action: Habits, norms, values, and rationality in electoral participation. Ration Soc. 2022;35(1):81–109. doi: 10.1177/10434631221142733

[2] Pattanaik PK. Voting and collective choice: Some aspects of the theory of group decision-making. Cambridge: Cambridge University Press; 1971.

[3] Weber RJ. Comparison of Public Choice Systems. Cowles Foundation for Research in Economics, Yale University. 1978. 498 p. Available from: https://elischolar.library.yale.edu/cowles-discussion-paper-series/732/

[4] Wolk S, Quinn J, Ogren M. STAR Voting, equality of voice, and voter satisfaction: considerations for voting method reform. Const Polit Econ. 2023;34(3):310–34. doi: 10.1007/s10602-022-09389-3

[5] Brams SJ, Kilgour DM. Satisfaction approval voting. Mathematical and Computational Modeling: With Applications in Natural and Social Sciences, Engineering, and the Arts. 2015. p. 273–98.

[6] Mann S, Wüstemann H. Efficiency and utility: an evolutionary perspective. Int J Soc Econ. 2010;37(9):676–85. doi: 10.1108/03068291011062470

[7] Scott J. Rational choice theory. Understanding contemporary society: Theories of the present. 2000. p. 126–38.

[8] Browning G, Webster F, Halcli A. Understanding contemporary society: Theories of the present. Sage; 1999.

[9] Boudon R. Beyond Rational Choice Theory. Annu Rev Sociol. 2003;29(1):1–21. doi: 10.1146/annurev.soc.29.010202.100213

[10] Stevens D. Thompson WR, editor. Satisficing in Political Decision Making. Oxford Research Encyclopedia of Politics. Oxford University Press; 2019. Available from: https://oxfordre.com/politics/display/10.1093/acrefore/9780190228637.001.0001/acrefore-9780190228637-e-1020

[11] Hibbing JR, Theiss-Morse E. Stealth democracy: Americans’ beliefs about how government should work. Cambridge University Press; 2002.

[12] Anderson CJ, Guillory CA. Political Institutions and Satisfaction with Democracy: A Cross-National Analysis of Consensus and Majoritarian Systems. Am Polit Sci Rev. 1997;91(1):66–81. doi: 10.2307/2952259

[13] Alshmemri M, Shahwan-Akl L, Maude P. Herzberg’s two-factor theory. Life Sci J. 2017;14(5):12–6.

[14] Schäfer A, Wenker J. Losers’ Dissent: How Election Results Shape Populists’ Satisfaction with Democracy. Government and Opposition. 2025. p. 1–21.

[15] Ezrow L, Xezonakis G. Satisfaction with democracy and voter turnout: A temporal perspective. Party Polit. 2016;22(1):3–14.

[16] Smith CA, Lazarus RS. Emotion and adaptation. Handbook of personality: Theory and research. 1990. p. 609–37.

[17] Pascual‐Nebreda L, Cabanelas‐Lorenzo P, Blanco‐González A. Understanding dissatisfaction through evaluation theory. Manage Decis Econ. 2022;43(7):3116–29. doi: 10.1002/mde.3585

[18] Pascual Nebreda L, Díez Martín F, Blanco González A. Changes and evolution in the intellectual structure of consumer dissatisfaction. J Consum Behav. 2020;20(1):160–72. doi: 10.1002/cb.1864

[19] Farrell DM, Mcallister I. Voter satisfaction and electoral systems: Does preferential voting in candidate‐centred systems make a difference? Eur J Polit Res. 2006;45(5):723–49. doi: 10.1111/j.1475-6765.2006.00633.x

[20] Saari DG. Basic geometry of voting. Berlin: Springer; 1995.

[21] Fishburn PC, Gehrlein WV. Borda’s rule, positional voting, and Condorcet’s simple majority principle. Public Choice. 1976;28(1):79–88. doi: 10.1007/bf01718459

[22] Nurmi H. Comparing voting systems. Dordrecht: Springer; 1987.

[23] Young HP. Condorcet’s theory of voting. Am Polit Sci Rev. 1988;82(4):1231–44.

[24] Menéndez ML, Pardo JA, Pardo L, Pardo MC. The Jensen-Shannon divergence. J Frankl Inst. 1997;334(2):307–18. doi: 10.1016/s0016-0032(96)00063-4

[25] Terzopoulou Z, Endriss U. Neutrality and relative acceptability in judgment aggregation. Soc Choice Welf. 2020;55(1):25–49. doi: 10.1007/s00355-019-01230-5

[26] Kamada Y, Kojima F. Voter Preferences, Polarization, and Electoral Policies. Am Econ J Microecon. 2014;6(4):203–36. doi: 10.1257/mic.6.4.203

[27] Barbaro S. Election methods and political polarization. Math Soc Sci. 2025;135:102417. doi: 10.1016/j.mathsocsci.2025.102417

[28] Nagtzaam MAM, van Erkel PFA. Preference votes without preference? Institutional effects on preference voting: an experiment. J Elections Public Opin Parties. 2017;27(2):172–91. doi: 10.1080/17457289.2016.1243542

[29] Green PE, Rao VR. Applied multidimensional scaling: A comparison of approaches and algorithms. 1972.

[30] Pathria RK, Beale PD. Statistical Mechanics. 3rd ed. Oxford: Academic Press; 2011.

[31] Galam S. Sociophysics: A review of Galam models. Int J Modern Phys C. 2008;19(03):409–40. doi: 10.1142/S0129183108012297

[32] Castellano C, Fortunato S, Loreto V. Statistical physics of social dynamics. Rev Mod Phys. 2009;81(2):591–646. doi: 10.1103/revmodphys.81.591

[33] List C. Zalta EN, Nodelman U, editors. Social Choice Theory. Metaphysics Research Lab, Stanford University; 2022. The Stanford Encyclopedia of Philosophy, Winter 2022 Edition. Available from: https://plato.stanford.edu/archives/win2022/entries/social-choice/

[34] Kullback S. Kullback-leibler divergence. Tech Rep. 1951.

[35] Murphy KP. Binomial and multinomial distributions. University of British Columbia, Tech Rep; 2006.

[36] Minka T. Estimating a Dirichlet Distribution. Massachusetts Institute of Technology; 2000.

[37] Lange K. Applications of the Dirichlet distribution to forensic match probabilities. Genetica. 1995;96(1–2):107–17. doi: 10.1007/BF01441156 7607447

[38] Emerson P. The original Borda count and partial voting. Soc Choice Welf. 2011;40(2):353–8. doi: 10.1007/s00355-011-0603-9

[39] Schulze M. A new monotonic, clone-independent, reversal symmetric, and condorcet-consistent single-winner election method. Soc Choice Welf. 2011;36(2):267–303. doi: 10.1007/s00355-010-0475-4

[40] Faliszewski P, Hemaspaandra E, Schnoor H. Copeland voting: Ties matter. In: Proceedings of the 7th international joint conference on Autonomous agents and multiagent systems-Volume 2. 2008. p. 983–90.

[41] Meir R. Plurality Voting under Uncertainty. In: Proceedings of the Twenty-Ninth AAAI Conference on Artificial Intelligence. Palo Alto (CA): The AAAI Press; 2015. p. 2103–9.

[42] Johnson RA, Wichern DW. Applied Multivariate Statistical Analysis. 5th ed. Upper Saddle River (NJ): Prentice Hall; 2002.

[43] Santos JM, Embrechts M. On the use of the adjusted rand index as a metric for evaluating supervised classification. In: International conference on artificial neural networks. Springer; 2009. p. 175–84.

[44] Black D. Partial justification of the Borda count. Public Choice. 1976;28(1):1–15. doi: 10.1007/bf01718454

[45] Young HP. An axiomatization of Borda’s rule. J Econ Theor. 1974;9(1):43–52. doi: 10.1016/0022-0531(74)90073-8

[46] Grofman B, Feld SL, Fraenkel J. Finding the Threshold of Exclusion for all single seat and multi-seat scoring rules: Illustrated by results for the Borda and Dowdall rules. Math Soc Sci. 2017;85:52–6. doi: 10.1016/j.mathsocsci.2016.11.004

[47] Grofman B, Feld SL, Fraenkel J. Finding the threshold of exclusion for all single seat and multi-seat scoring rules: illustrated by results for the Borda and Dowdall rules. Math Soc Sci. 2017;85:52–6.

[48] Tideman N. The Single Transferable Vote. J Econ Perspect. 1995;9(1):27–38. doi: 10.1257/jep.9.1.27

[49] Gunantara N. A review of multi-objective optimization: Methods and its applications. Cogent Eng. 2018;5(1):1502242. doi: 10.1080/23311916.2018.1502242

[50] Duclos JY, Esteban J, Ray D. Polarization: concepts, measurement, estimation. In: The social economics of poverty. Routledge; 2006. p. 54–102.

[51] Wolfson MC. When inequalities diverge. Am Econ Rev. 1994;84(2):353–8.

[52] Deeb OE. Entropic spatial auto-correlation of voter uncertainty and voter transitions in parliamentary elections. Phys A: Stat Mech Appl. 2023;617:128675. doi: 10.1016/j.physa.2023.128675

[53] Schwartz B, Ward A, Monterosso J, Lyubomirsky S, White K, Lehman DR. Maximizing versus satisficing: happiness is a matter of choice. J Pers Soc Psychol. 2002;83(5):1178–97. doi: 10.1037/0022-3514.83.5.1178 12416921

[54] Reutskaja E, Lindner A, Nagel R, Andersen RA, Camerer CF. Choice overload reduces neural signatures of choice set value in dorsal striatum and anterior cingulate cortex. Nat Hum Behav. 2018;2(12):925–35. doi: 10.1038/s41562-018-0440-2 30988434

[55] Hemaspaandra E, Spakowski H, Vogel J. The complexity of Kemeny elections. Theor Comput Sci. 2005;349(3):382–91. doi: 10.1016/j.tcs.2005.08.031

[56] Mukhopadhyay A, Mazumdar RR, Roy R. Voter and Majority Dynamics with Biased and Stubborn Agents. J Stat Phys. 2020;181(4):1239–65. doi: 10.1007/s10955-020-02625-w

[57] Hawthorne F, Harunari PE, de Oliveira MJ, Fiore CE. Nonequilibrium Thermodynamics of the Majority Vote Model. Entropy (Basel). 2023;25(8):1230. doi: 10.3390/e25081230 37628260 PMC10453243

