# Supplementary material for: ElecMeter: An index for evaluating dissatisfaction of social decision-making
Source: PLoS One. 2026 Sep 11;21(9):e0354798. doi: 10.1371/journal.pone.0354798 (PMC13568507; doi:10.1371/journal.pone.0354798)
Supplement: Supplementary file 1 [file pone.0354798.s001.pdf]

Supplementary of
`ElecMeter`: an index for Evaluating an Election social Dissatisfaction

Maryam Shahdoust[1*], Zahra Eidi[1], Rouzbeh Torabi[1,3], and Mehdi Sadeghi[2]

[1]School of Biological Sciences, Institute For Research In Fundamental Sciences(IPM), Tehran, Iran
[2]Department of Medical Genetics,National Institute for Genetic Engineering and Biotechnology,Tehran,Iran
[3]Pegah Dadekavan Sharif Company (TAPSELL), Tehran, Tehran, IRAN
[*]Corresponding Author: m.shahdoost@ipm.ir

1 Borda Count

The Borda count is a positional voting method used to aggregate ranked preferences into a collective decision[1, 2, 3]. It assigns points to candidates based on their position in each voter's ranking. Specifically, in an election with K candidates, a candidate receives $K-1$ points for each first-place vote, $K-2$ points for each second-place vote, and so on, down to 0 points for a last-place vote.
Formally, for a voter i, let their ranked list of candidates be $c_1 \succ c_2 \succ \cdots \succ c_K$. Then, the Borda score assigned to candidate c_j by this voter is:$\text{Score}_i(c_j) = K - r_{ij}$, where r_{ij} is the rank of candidate c_j in voter i's list (with 1 being the highest rank).
The total Borda score for each candidate is the sum of their scores across all voters:
$\text{TotalScore}(c_j) = \sum_{i=1}^{N} \text{Score}_i(c_j)$.
The candidate with the highest total score is declared the winner.

2 Clustering Methodology

To identify patterns in the structure of dissatisfaction distributions across simulated populations, we applied hierarchical clustering using the Jensen–Shannon Divergence (JSD)[4, 5] as the distance metric. JSD provides a symmetric and bounded measure of divergence between probability distributions, making it well-suited for comparing voter dissatisfaction profiles. For each pair of distributions, we computed the JSD and constructed a distance matrix. We then performed agglomerative hierarchical clustering using the complete linkage method[6], which merges clusters based on the maximum pairwise distance. This approach emphasizes compact and well-separated clusters. The resulting dendrogram(Fig S1) was cut to form four distinct clusters, based on visual inspection and stability of the clustering structure. These clusters reflect qualitatively different patterns of voter sentiment, such as concentrated satisfaction, polarization, or extreme dissatisfaction. In addition, hierarchical clustering was applied separately to populations based on their average dissatisfaction (S) and the `ElecMeter` values. In these cases, the distance matrix was calculated using Euclidean distance between the respective values for each population. The number of clusters was again set to four to enable direct comparisons across the different clustering criteria. The dendrograms for each clustering approach are shown in the figure below.

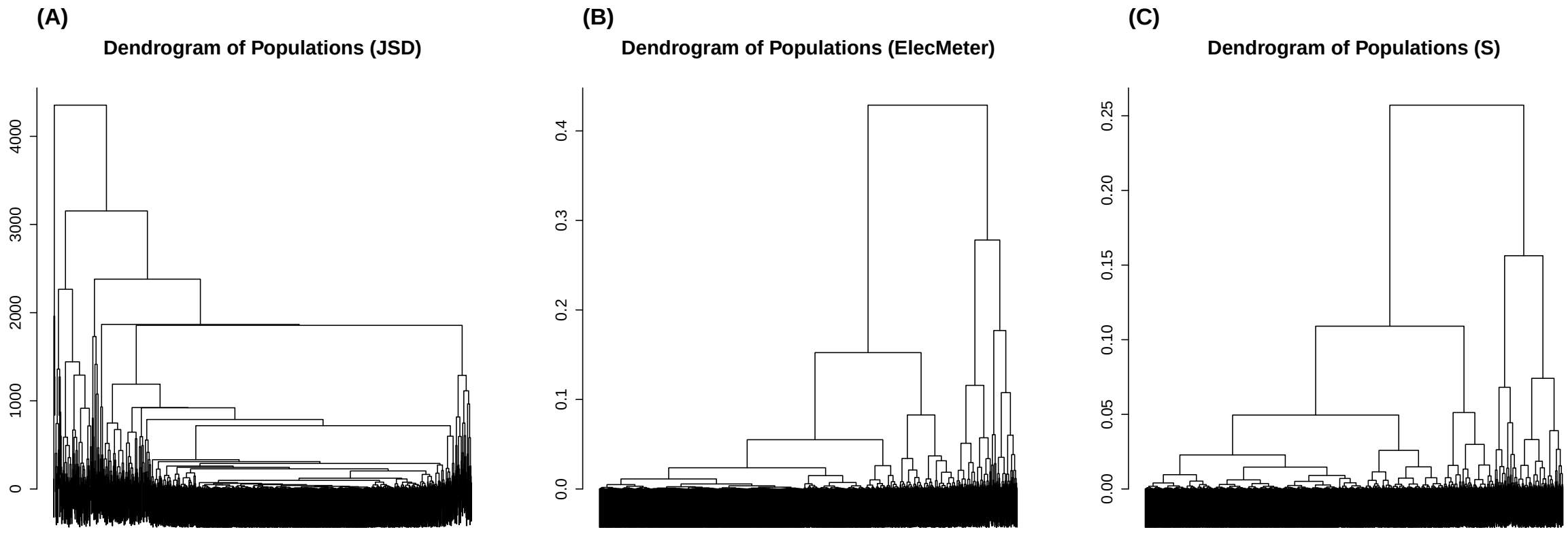

Figure S1: Dendrograms showing hierarchical clustering of simulated populations based on different measures of voter dissatisfaction. (A) Clustering using the Jensen–Shannon Divergence (JSD) between full dissatisfaction distributions. (B) Clustering based on normalized average dissatisfaction (S) using Euclidean distance. (C) Clustering based on the `ElecMeter` index using Euclidean distance. All dendrograms were cut to form four clusters, highlighting distinct patterns of voter sentiment, including concentrated satisfaction, polarization, and extreme dissatisfaction.

3 Sensitivity to Aggregation Rule

We evaluated alternative ways of aggregating the normalized divergences (I_1, I_2, I_3) into a single structural dissatisfaction component. In addition to the maximum operator ($I_{\max}$), we computed the arithmetic mean, the geometric mean, and the range $|\max(I_1, I_2, I_3) - \min(I_1, I_2, I_3)|$. These alternatives are denoted by I_{mean}, I_G, and I_{range}, respectively. Each variant yields values bounded within $[0, 1]$.

To assess robustness, we compared ElecMeter values across all simulated populations with different numbers of candidates ($K = 3, 4, 5, 7$), using both Pearson and Spearman correlations. All indices were constructed using Equation 11 in the main text, with $I_{\max}$ replaced by I_{mean}, I_G, or I_{range}. The resulting indices are denoted `ElecMeter_mean`, `ElecMeter_G`, and `ElecMeter_range`, respectively.

The results (Table S1) show that ElecMeter based on $I_{\max}$ is highly correlated with the mean- and geometric-based variants across all K. The range-based variant is less consistent, particularly in rank correlation for $K = 7$ ($\rho \approx 0.64$), reflecting its greater sensitivity to noise. These findings indicate that ElecMeter is robust to the choice of aggregation rule, while the use of $I_{\max}$ provides a conservative minimax approach that ensures sensitivity to extreme divergence patterns. All results in Table S1 are based on $p = 3$ in the index equation.
As shown in Table S1, the different ElecMeter aggregation rules are highly correlated across all K values. **"The results of the alternative indices for different simulations with varying numbers of candidates are available in the sensitivity aggregation section on GitHub, in addition to the Supplementary File."**

Table S1. Correlations between `ElecMeter` variants based on different aggregation rules.

K	Comparison	Pearson r	Spearman ρ
3	Max vs. Geometric mean	0.991	0.978
	Max vs. Arithmetic mean	0.994	0.979
	Max vs. Range	0.996	0.981
4	Max vs. Geometric mean	0.991	0.954
	Max vs. Arithmetic mean	0.995	0.964
	Max vs. Range	0.994	0.934
5	Max vs. Geometric mean	0.986	0.945
	Max vs. Arithmetic mean	0.990	0.957
	Max vs. Range	0.986	0.861
7	Max vs. Geometric mean	0.920	0.919
	Max vs. Arithmetic mean	0.938	0.931
	Max vs. Range	0.836	0.640

K is the number of candidates. "Max" denotes $I_{\max}$; "Geometric mean" and "Arithmetic mean" refer to the geometric and arithmetic means of (I_1, I_2, I_3); "Range" is the absolute difference between the maximum and minimum of (I_1, I_2, I_3). Values are rounded from analysis output.

4 Summary statistics for clusters for `ElecMeter` with different p

Table S2. Summary statistics for clusters based on the `ElecMeter` index

p	**Cluster**	**Median**	**Q1**	**Q3**	**IQR**
2	1	0.764	0.721	0.777	0.056
2	2	0.247	0.152	0.409	0.257
2	3	0.570	0.539	0.587	0.048
2	4	0.468	0.365	0.533	0.168
3	1	0.804	0.762	0.816	0.053
3	2	0.265	0.161	0.426	0.264
3	3	0.596	0.565	0.615	0.050
3	4	0.488	0.382	0.559	0.177
4	1	0.835	0.794	0.846	0.052
4	2	0.277	0.169	0.437	0.268
4	3	0.616	0.585	0.637	0.052
4	4	0.504	0.396	0.579	0.184

p is the value of the power parameter. Q1 and Q3 denote the first and third quartiles of `ElecMeter` values within each cluster, respectively. IQR denotes the interquartile range (Q3–Q1).

5 Boxplots of the ElecMeter index across clusters based on normalized average dissatisfaction (S)

To examine the distribution of ElecMeter values across clusters, we applied hierarchical clustering using the normalized average dissatisfaction (S).

Since the ElecMeter index includes a tunable power parameter p, selecting an appropriate value is important for balancing sensitivity to dissatisfaction extremes with robustness to distributional variation. To guide this choice, we computed ElecMeter values for $p \in \{2, 3, 4\}$ and examined their distributions across the JSD-based clusters (results shown in the main manuscript). Based on those results, we selected $p = 3$ as the primary parameter setting.

To validate this choice, we further examined the distribution of ElecMeter values across clusters obtained from S-based hierarchical clustering (Fig S2). Across all three parameter settings, the relative ordering of median ElecMeter values among clusters was consistent, with Cluster 1 showing the highest values and Cluster 4 the lowest. The separation between intermediate clusters was similar for $p = 3$ and $p = 4$, with $p = 3$ providing the best balance between discrimination of structural patterns and interpretability. Therefore, $p = 3$ was selected as the default setting for subsequent analyses.

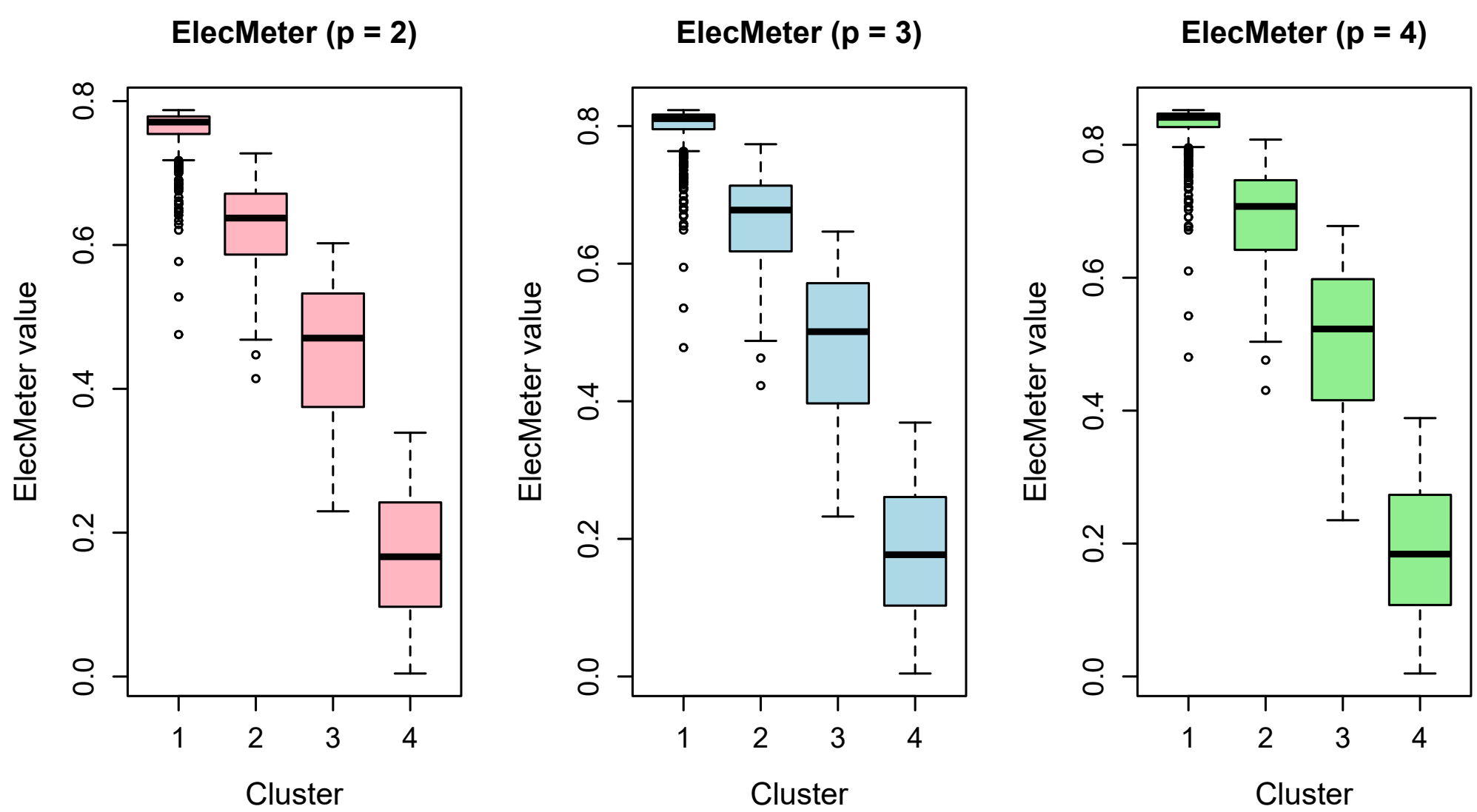

Figure S2: Boxplots of the ElecMeter index across four clusters identified using normalized average dissatisfaction (S). Results are shown for different values of the power parameter $p \in \{2, 3, 4\}$, with normalization by $2^{1/p}$. While average dissatisfaction is held fixed within clusters, the distributions of ElecMeter values differ across clusters, indicating sensitivity to structural differences in dissatisfaction profiles beyond mean levels alone. Higher values correspond to distributions with greater divergence from reference dissatisfaction patterns.

6 Breakfast Items Dataset

The breakfast dataset originates from the study by Green and Rao (1972)[7]. In this study, 42 respondents (21 Wharton MBA students and their wives) were asked to provide strict preference rankings over 15 sweet breakfast items. Preferences were elicited under six different contexts, such as "Overall preferences" and "When I'm having a breakfast consisting of juice, bacon and eggs, and beverage." The rankings for each context are provided in separate data files within the PrefLib collection. In our analysis, we focused on the "Overall preferences" dataset.

At first, we consider each item as the winning candidate. For each item, dissatisfaction distributions were computed and the corresponding ElecMeter indices were calculated for parameter values $p = 2, 3, 4$, and 5. The results consistently identified one candidate (" Danish pastry") as having the lowest dissatisfaction across all participants, with index values ranging from 0.564 ($p = 2$) to 0.651 ($p = 5$). In contrast, item "Hard rolls and butter"produced the highest ElecMeter indices (0.802–0.852), indicating that it was the least representative option. The results are in Table S3. In addition, the total results including the dissatisfaction distributions for all the items are in "Breakfast_total_results" in GitHub.

Table S3. ElecMeter indices and dissatisfaction metrics for breakfast items.

**Breakfast item**	**Norm. avg. diss.**	$I_{\max}$	**ElecMeter**$(p=2)$	**ElecMeter**$(p=3)$	**ElecMeter**$(p=4)$	**ElecMeter**$(p=5)$
Toast pop-up	0.765	0.811	0.789	0.789	0.789	0.790
Buttered toast	0.495	0.876	0.712	0.735	0.755	0.772
English muffin & margarine EMM	0.456	0.949	0.744	0.780	0.808	0.830
Jelly donut	0.534	0.857	0.714	0.731	0.747	0.760
Cinnamon toast	0.517	0.929	0.752	0.778	0.800	0.817
Blueberry muffin & margarine	0.378	0.885	0.680	0.720	0.750	0.773
Hard rolls & butter	0.599	0.961	0.801	0.820	0.837	0.852
Toast & marmalade	0.536	0.900	0.740	0.761	0.779	0.795
Buttered toast & jelly	0.463	0.911	0.723	0.753	0.779	0.799
Toast & margarine	0.643	0.882	0.772	0.781	0.789	0.797
Cinnamon bun	0.424	0.890	0.697	0.731	0.758	0.779
Danish pastry	0.281	0.747	0.564	0.603	0.631	0.651
Glazed donut	0.468	0.882	0.706	0.734	0.756	0.775
Coffee cake	0.299	0.836	0.628	0.674	0.706	0.729
Corn muffin & butter	0.645	0.860	0.760	0.767	0.775	0.781

Norm. avg. diss. denotes the normalized average dissatisfaction. $I_{\max}$ represents the maximum of $\{I_1, I_2, I_3\}$. The columns corresponding to the `ElecMeter` denote the `ElecMeter` index computed using power parameter $p = \{2, 3, 4, 5\}$.

To assess robustness, we calculated correlations between indices obtained with different p values. The results demonstrated consistently high correlations (r = 0.79–0.96), showing that the relative ranking of candidates remains stable across parameter choices. Notably, the correlation between $p = 4$ and $p = 5$ was the strongest ($r = 0.96$), while the correlation between $p = 2$ and $p = 5$ was weaker but still high ($r = 0.79$). Importantly, all `ElecMeter` values were above 0.5, suggesting that no single candidate generated broad consensus among voters. Even the best-performing candidate left participants moderately dissatisfied, reflecting the diversity of preferences in the dataset.

Table S4. Spearman correlations between `ElecMeter` indices computed using different power parameters for the Breakfast Items dataset.

	ElecMeter($p = 2$)	ElecMeter($p = 3$)	ElecMeter($p = 4$)	ElecMeter($p = 5$)
ElecMeter($p = 2$)	1	0.9750	0.8679	0.7929
ElecMeter($p = 3$)	0.9750	1	0.9286	0.8500
ElecMeter($p = 4$)	0.8679	0.9286	1	0.9643
ElecMeter($p = 5$)	0.7929	0.8500	0.9643	1

Values represent Spearman rank correlation coefficients between `ElecMeter` indices computed using power parameters $p \in \{2, 3, 4, 5\}$.

References

[1] Peter Emerson. The original borda count and partial voting. *Social Choice and Welfare*, 40(2):353–358, 2013.

[2] Peter C Fishburn and William V Gehrlein. Borda's rule, positional voting, and condorcet's simple majority principle. *Public Choice*, pages 79–88, 1976.

[3] Duncan Black. Partial justification of the borda count. *Public Choice*, pages 1–15, 1976.

[4] María Luisa Menéndez, Julio Angel Pardo, Leandro Pardo, and María del C Pardo. The jensen-shannon divergence. *Journal of the Franklin Institute*, 334(2):307–318, 1997.

[5] Solomon Kullback. Kullback-leibler divergence. *Tech. Rep.*, 1951.

[6] Richard Arnold Johnson and Dean W Wichern. *Applied Multivariate Statistical Analysis.* Prentice Hall, Upper Saddle River, NJ, 5th edition, 2002.

[7] Paul E Green and Vithala R Rao. Applied multidimensional scaling: A comparison of approaches and algorithms. *(No Title)*, 1972.
